# SUPER Scheme in Action: Experimental Demonstration of Red-Detuned Excitation of a Quantum Emitter

**DOI:** 10.1021/acs.nanolett.2c01783

**Published:** 2022-07-06

**Authors:** Yusuf Karli, Florian Kappe, Vikas Remesh, Thomas K. Bracht, Julian Münzberg, Saimon Covre da Silva, Tim Seidelmann, Vollrath Martin Axt, Armando Rastelli, Doris E. Reiter, Gregor Weihs

**Affiliations:** † Institut für Experimentalphysik, Universität Innsbruck, Innsbruck 6020, Austria; ‡ Institut für Festkörpertheorie, Universität Münster, Münster 48149, Germany; § Institute of Semiconductor and Solid State Physics, Johannes Kepler University Linz, Linz 4040, Austria; ∥ Theoretische Physik III, Universität Bayreuth, Bayreuth 95440, Germany

**Keywords:** off-resonant, quantum dot, pulse shaping, single photon, coherent control

## Abstract

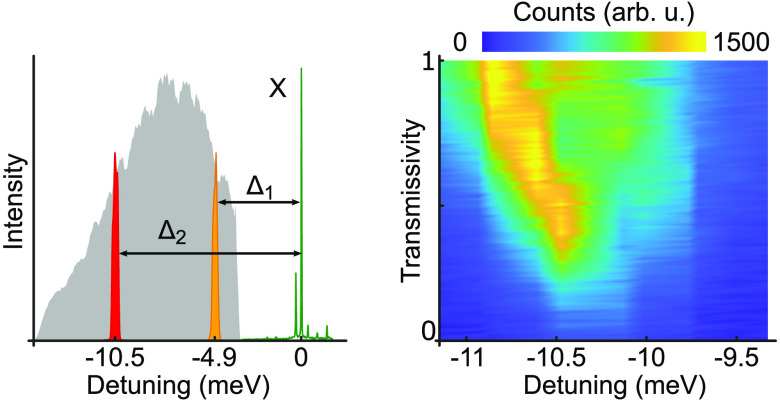

The quest for the perfect single-photon source includes finding the optimal protocol for exciting the quantum emitter. Coherent optical excitation was, up until now, achieved by tuning the laser pulses to the transition frequency of the emitter, either directly or in average. Recently, it was theoretically discovered that an excitation with two red-detuned pulses is also possible where neither of which would yield a significant upper-level population individually. We show that the so-called swing-up of quantum emitter population (SUPER) scheme can be implemented experimentally with similar properties to existing schemes by precise amplitude shaping of a broadband pulse. Because of its truly off-resonant nature, this scheme has the prospect of powering high-purity photon sources with superior photon count rate.

The future of photonic quantum technologies relies on bright, photostable, and on-demand sources of single and indistinguishable photons. To achieve the on-demand character, a deterministic state preparation of the excited state is required. The most prominent scheme for coherent optical control is the Rabi scheme, where a laser pulse tuned to the transition energy, called the π-pulse, inverts the quantum emitter population. As soon as the laser energy is detuned, the inversion fidelity drops drastically.^1^ Consequently, all coherent excitation schemes to achieve a high population inversion either have a frequency component at^2−7^ or are in average^8,9^ resonant with the transition frequency of the quantum emitter. From this picture, a truly off-resonant excitation scheme is not desirable, even though these have the advantage of requiring only spectral filtering instead of challenging methods based on polarization filtering. Surprisingly, it was recently theoretically demonstrated that there exists a swing-up mechanism in the so-called Swing-UP of quantum EmitteR population (SUPER) scheme.^10^ The SUPER scheme relies on the coherent coupling of two red-detuned lasers to coherently excite the emitter, while an individual pulse would not lead to any upper-level occupation. Up until now, this scheme existed only as a theoretical possibility. In this Letter, we show that the SUPER scheme works in a simple, yet elegant experiment to excite a quantum emitter, relying on amplitude-shaping of a broadband laser pulse.

As the quantum emitters, we choose semiconductor quantum dots because they have emerged as a promising platform for quantum communication devices with excellent performance characteristics.^11−20^ Quantum dots benefit from their excellent photostability, nearly Fourier-limited emission line width and growth technologies that allow easy integration into nanoscale devices.^21−25^

Our results prove that the SUPER scheme is an efficient method to excite a quantum dot to its excited state with the same efficiency as the Rabi scheme. The off-resonant nature of this excitation bears the potential for a wide range of applications where a resonant excitation should be avoided, in particular for using quantum dots as photon sources.

We start by briefly summarizing the idea of the SUPER scheme.^10^ We consider a quantum dot as a two-level system consisting of ground |*g*⟩ and exciton state |*x*⟩, separated by an energy *ℏω*_0_. This system is driven by a pulsed laser encoded in the time-dependent term Ω(*t*). Within the dipole and rotating wave approximations, the Hamiltonian for this system reads
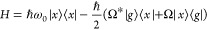
1The SUPER scheme requires two laser pulses that are both red-detuned (Δ_1_ and Δ_2_) from the exciton state by several millielectronvolts. In the following, we will always refer to the pulse with the smaller detuning as first pulse, that is, |Δ_1_| < |Δ_2_|. Considering a Gaussian-shaped excitation pulse, we define the generalized Rabi frequency as , where Ω_*i*_ is the resonant Rabi frequency of either pulse given at the maximum of the pulse temporal envelope. In the SUPER scheme, one achieves a gradual rise in the exciton population by modulating the Rabi frequency, through the beating of the two detuned pulses. If the difference between the two detunings coincides with the Rabi frequency of the first pulse, that is,, implying the condition |Δ_2_| > 2|Δ_1_|, the SUPER mechanism results in a complete population inversion of the quantum emitter.^10^

The experimental implementation of the SUPER scheme relies on frequency-domain amplitude shaping. In the calculation, instead of specifying the single laser pulse parameters explicitly, Ω(*t*) is obtained by an inverse Fourier transform of the laser spectrum that is multiplied by an amplitude mask to describe the pulse shaping process. Starting point is a Gaussian frequency spectrum
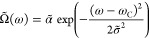
2with a spectral full width at half maximum (fwhm) of , centered around a detuning of *ℏ*(ω_*C*_ – ω_0_) = −8.4 meV and an integrated resonant pulse area of α̃ = 26π. The amplitude function is approximated by two normalized Gaussian functions
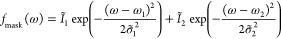
3

This results in two contributions to the resulting spectrum at ω_*i*_ with variable transmission *Ĩ*_*i*_. From this, we define the detunings as Δ_*i*_ = *ℏ*(ω_*i*_ – ω_0_). The spectral width  is chosen to 0.2 meV for all calculations. To account for experimental imperfections of the pulse-shaping process, a transmission of 5% between the two peaks is added. Note that the transmission *Ĩ*_*i*_ is for the electric field in contrast to the experimental scenario where *I*_*i*_ (called transmissivity) is the intensity, that is, *I*_i_ corresponds to (*Ĩ*_i_)^2^. A representative amplitude-shaped spectrum of the intensity is shown in Figure 1a. To calculate the exciton occupation, we derive the equations of motion from the Hamiltonian using the von-Neumann equation, which is then numerically integrated.^10^ Because the phonon influence on the SUPER scheme has been shown to be weak,^26^ we neglect phonons in the present calculations. In Figure 1b, we show an exemplary time dynamics at Δ_1_ = –4.9 meV, Δ_2_ = −11.12 meV and *I*_1_ = 0.5, *I*_2_ = 0.96.

**Figure 1:**
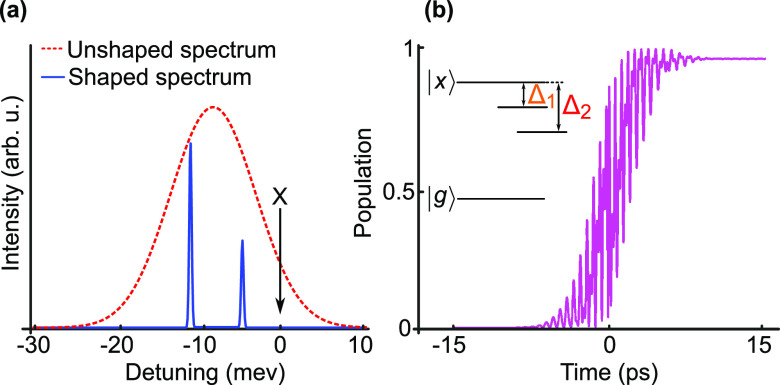
Concept of SUPER scheme: (a) Spectrum of the broadband excitation pulse (red dashed curve) and an exemplary pulse pair (blue solid curve) after spectral shaping with transmissivities *I*_1_ = 0.5 and *I*_2_ = 0.96 at detunings Δ_1_ = −4.9 meV and Δ_2_ = −11.12 meV. Black arrow denotes the position of the targeted exciton state. (b) Calculated dynamics of the exciton population using the shaped spectrum. Inset shows the energy level scheme with detunings. |g⟩ , ground state, |x⟩, exciton state.

To experimentally obtain the two red-detuned pulses with appropriate detunings, we implement frequency-domain amplitude shaping with a folded 4*f* pulse shaper equipped with a programmable spatial light modulator (SLM, CRi, 128 pixels). The experimental setup is summarized in Figure 2. A broadband Ti:sapphire laser (MIRA 900, Coherent) produces 120 fs long, Gaussian-shaped pulses with the central wavelength of 802 nm, pulse energy of ∼4 nJ and a peak power of ∼12 kW. The collimated laser beam that enters the 4*f* pulse shaper is first dispersed by a blazed diffraction grating (1800 lines/mm, Newport), and then focused onto the SLM with a curved mirror (*f* = 500 mm), such that each pixel is assigned a narrow laser spectral window of ∼0.09 nm (0.17 meV in energy, for details see SI). The amplitude-shaped laser beam travels the same path back and leaves the pulse shaper toward the cryostat with the quantum dot. The inset in Figure 2 shows a representative amplitude-shaped spectrum.

**Figure 2:**
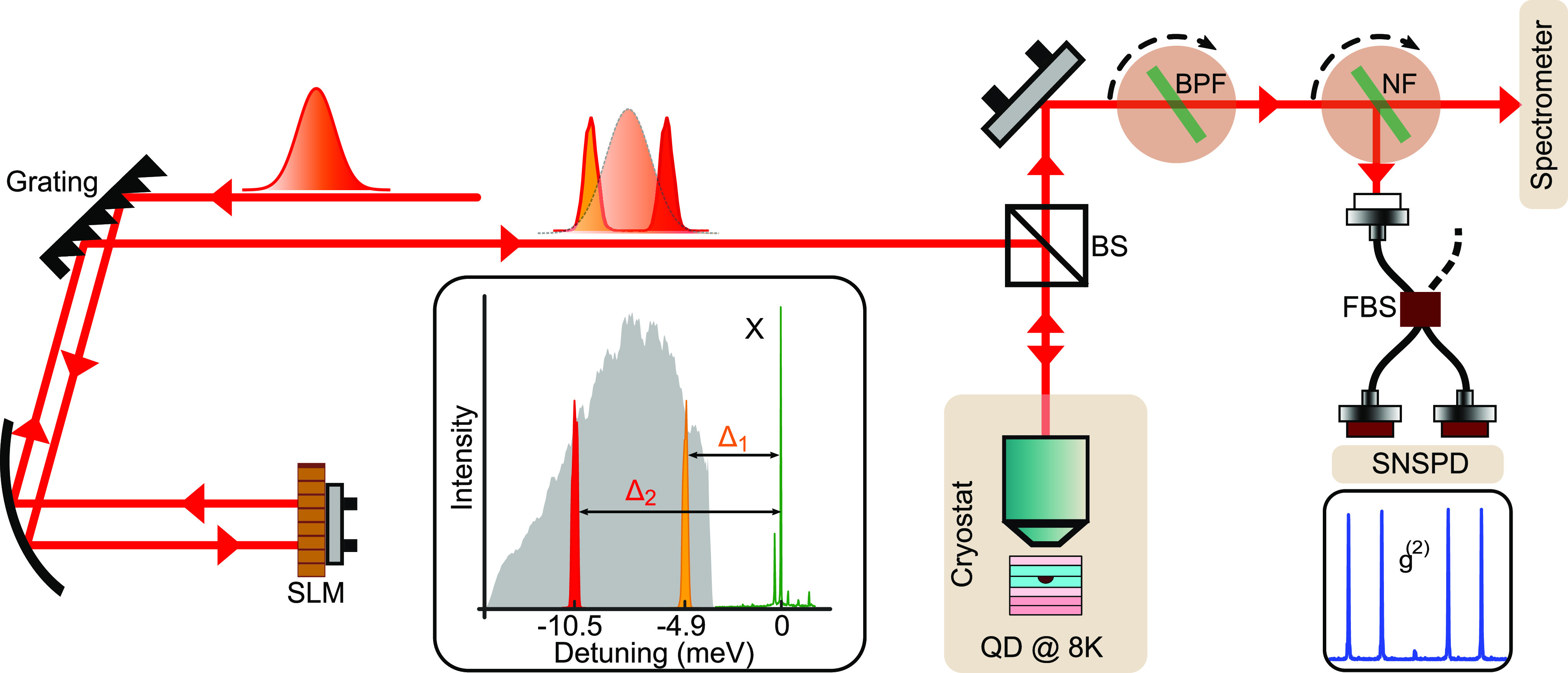
Sketch of the experimental setup: The laser beam is guided to a folded 4*f* pulse shaper equipped with a spatial light modulator (SLM) for amplitude-shaping the broadband spectrum (gray shade, inset). Incoming and outgoing beams are shown as separate paths for clarity. The shaped pulse-pair is directed with a beam splitter (BS) to the cryostat that holds the quantum dot at 8 K. Emitted photons from the quantum dot are sent through a bandpass filter (BPF) and a notch filter (NF) to either the spectrometer or with an additional fiber beam splitter (FBS) to the superconducting nanowire single-photon detectors (SNSPD) to record the photon coincidences. On the basis of above-band excitation, the quantum dot exciton emission line (X, green) is identified. An exemplary pulse-pair with detunings Δ_1_ (orange) and Δ_2_ (red) is also shown.

To characterize the detunings of the two pulses with respect to the exciton line, we first performed above-band gap excitation of the quantum dot. The resulting emission spectrum is shown as a green curve in Figure 2. A sharp exciton-emission line (X) is identified at 798.66 nm, surrounded by phonon sidebands and substrate emission. Based on this, we can choose the detunings Δ_1_ and Δ_2_. Figure 2 also shows the unshaped laser spectrum as gray-shaded area. Its sharp edge on the high energy sideis due to a razor blade mounted behind the SLM to suppress the laser spectral tail that is resonant with the quantum dot emission line. The intensities of both pulses can be tuned individually by varying the transmissivities of the SLM pixels from 0 to 1, denoted as *I*_1_ and *I*_2_. For our experiments, the intensities of the second pulse at Δ_2_ = −10.6 meV range from 0.7 μW (*I*_2_ = 0) to 18.8 μW (*I*_2_ = 1) while the first pulse intensity *I*_1_ was fixed to 15.5 μW, measured at the cryostat window.

Our sample consists of GaAs/AlGaAs quantum dots obtained by the Al-droplet etching method.^27,28^ The dots are embedded in the center of a λ-cavity placed between a bottom (top) distributed Bragg reflector consisting of 9(2) pairs of λ/4 thick Al_0.95_Ga_0.05_As/Al_0.2_Ga_0.8_As layers. The sample is kept in a closed-cycle cryostat with base temperature 8 K on a three-axis piezoelectric stage (ANPx101/ANPz102, attocube systems AG). The shaped pulse pair is focused onto the quantum dot with a cold aspheric lens (NA = 0.77, Edmund Optics) and the emission is collected via the same path backward, through a combination of a bandpass filter (808 nm, fwhm 3 nm, Layertec) and a notch filter (BNF-805-OD3, fwhm 0.3 nm, Optigrate) to a single-photon sensitive spectrometer (Acton SP-2750, Roper Scientific) equipped with a liquid nitrogen-cooled charge-coupled device camera (Spec10 CCD, Princeton Instruments) or superconducting nanowire single-photon detectors (SNSPD, Eos, Single Quantum). For estimating the wavelength-independent background, we integrate the photon counts on the high-energy sideband of the exciton emission peak (for a detailed discussion, see Figure S1 in SI).

To measure the SUPER scheme, we fix the detuning and the transmissivity of the first pulse to Δ_1_ = −4.9 meV and *I*_1_ = 0.5, respectively. We then vary the transmissivity of the second pulse (*I*_2_), and record the emitted spectra for various detunings (Δ_2_). The results are displayed as a two-dimensional map in Figure 3a, as a function of Δ_2_ and *I*_2_, where the color scale denotes the integrated photon counts. Every automated transmissivity scan (that is, individual columns in Figure 3a) records emitted spectra for 100 different *I*_2_ values, and the experiment is performed for 11 different Δ_2_ values. All the data shown are background-corrected as described in Figure S1 in SI. At zero intensity of the second pulse, that is, *I*_2_ = 0, only negligible photon counts are recorded, implying that no excitation occurs in the absence of the second pulse, even if the first pulse is present. By increasing *I*_2_ from 0 to 1, the exciton counts gradually increase, specifically for detunings around Δ_2_ = −10 to −11 meV. We find a clear region of high photon counts demonstrating that the exciton state becomes occupied by the two-pulse excitation. To validate further that the exciton state only gets populated when both pulses are switched on, we set *I*_1_ = 0, and perform the *I*_2_ – Δ_2_ scan, as in Figure 3a, which does not result in any significant exciton emission (see Figure S3a in SI).

**Figure 3:**
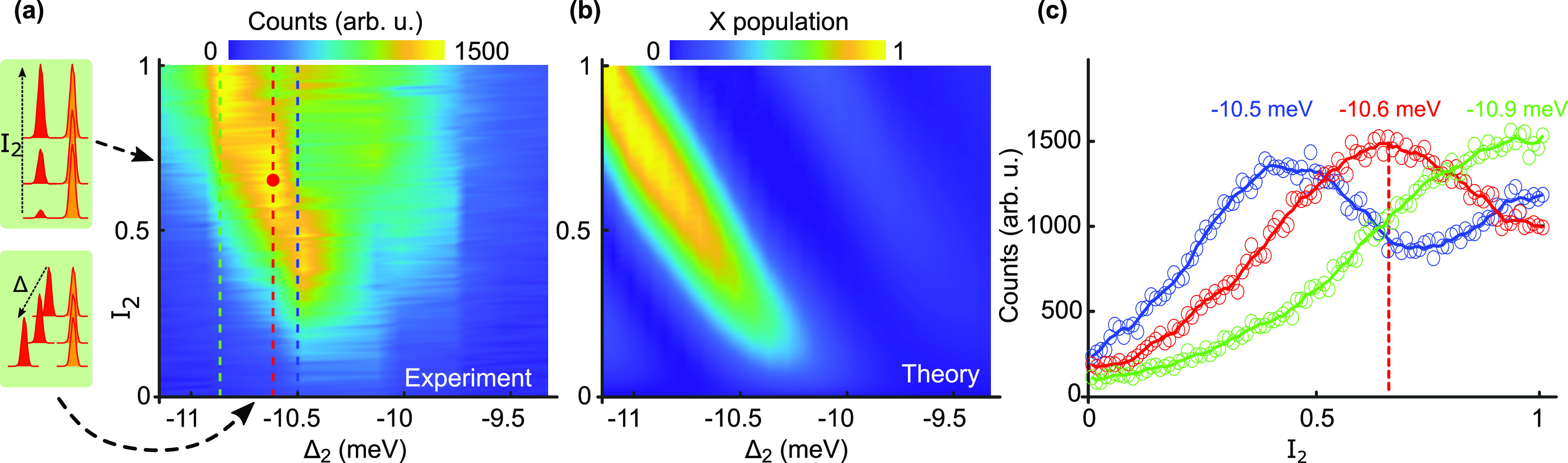
Exciton population achieved by the SUPER scheme: (a) Measured photon counts at exciton emission energy as a function of the detuning Δ_2_ and the transmissivity *I*_2_ of the second pulse. The first pulse is fixed to Δ_1_ = −4.9 meV and *I*_1_ = 0.5. The scale shows the integrated exciton counts after correcting for background. The red dot indicates the parameters used in the photon quality experiment (Figure 4). (b) Theoretically calculated exciton (X) population based on a two-level system. (c) Vertical line-cuts through the 2D map for Δ_2_ = −10.5 meV (blue), –10.6 meV (red), and −10.9 meV (green). The dashed red line indicates the parameters at which the photon quality measurements are performed.

Therefore, we conclude that under the action of two pulses below the absorption edge of the quantum dot an excitation via the SUPER scheme has taken place. The calculated dynamics of the two-level system under the red-detuned two-pulse excitation is shown in Figure 3b. The experimentally observed high exciton occupation at Δ_2_ ≈ −10.5 meV with a diagonal trend toward larger Δ_2_ and *I*_2_, shows excellent agreement with the theoretical results. The calculated maximum exciton occupation is ∼97% in the considered parameter window. For both experiment and theory, the condition that |Δ_2_| > 2|Δ_1_| holds.

Figure 3c shows line plots of the measured exciton occupation for Δ_2_ = −10.5, −10.6, and −10.9 meV featuring another interesting behavior: for the largest detuning Δ_2_ = −10.9 meV (green line), we find that the exciton counts increase monotonically with increasing *I*_2_. For Δ_2_ = −10.6 meV (red line), we find an increase in exciton counts up to *I*_2_ = 0.64, after which it decreases again. The most striking observation is for Δ_2_ = −10.5 meV (blue line), which shows close to 1.5 oscillations from *I*_2_ = 0–1 with a maximum at *I*_2_ = 0.4 and a minimum at *I*_2_ = 0.7. All these findings provide compelling evidence that the recorded exciton emission is due to the coherent excitation with two red-detuned pulses.

Following the experimental verification, here we discuss how the SUPER scheme compares to other schemes and its scope for quantum technologies. While a detailed comparison with all existing schemes goes beyond the scope of this paper, we investigate a different dot in the same sample under resonant two-photon-excitation (TPE) and SUPER excitation conditions. To perform TPE, we tune the excitation wavelength to the biexciton transition by shifting the amplitude mask in the SLM. The integrated photon counts at the exciton-emission energy obtained by the TPE (Figure 4a, blue circles) show coherent Rabi oscillation, as has been observed in similar works.^29^ Most importantly, the maxima of both oscillations coincide, clearly demonstrating that SUPER reaches the same efficiency as TPE. Furthermore, in Figure 4b we show the emission spectra of the quantum dot under TPE (top panel) and SUPER excitation (bottom panel). The TPE spectrum shows the exciton and biexciton emission peaks in addition to the scattered laser energy, while the SUPER spectrum shows the exciton emission peak and the first detuned pulse. As expected, the exciton emission lines in both spectra coincide. Notably, the first detuned pulse in SUPER is clearly distant from the biexciton energy and has no chance of exciting any transition other than the targeted exciton state.

**Figure 4:**
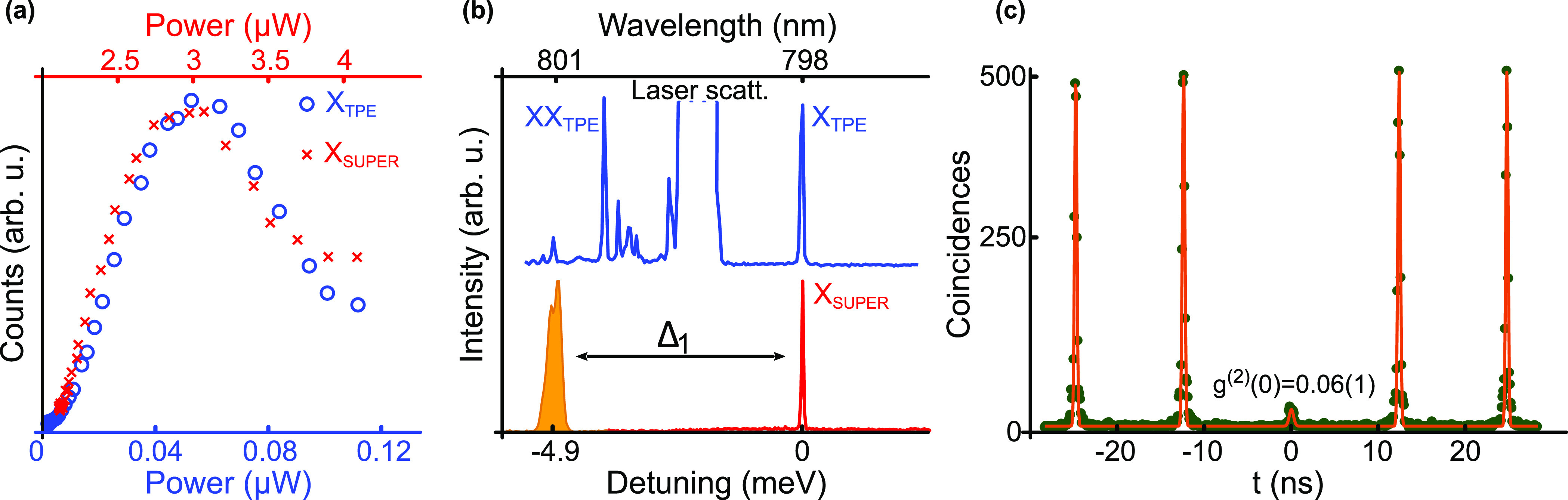
Scope of SUPER scheme: (a) Measured photon counts at the exciton-emission energy (red crosses) under SUPER excitation in contrast to resonant TPE (blue circles). The *x*-axes values show corresponding power measured with a 1% beam sampler near the cryostat window. (b) Quantum dot emission spectra under TPE (top panel) and SUPER excitation (bottom panel). The TPE emission spectrum shows residual laser scattering and the SUPER emission spectrum shows the first detuned pulse. (c) Single photon characteristics under SUPER excitation as shown by *g*^(2)^(0) = 0.06(1). The dark green dots show the measured data, while the orange curves denote the fit.

Furthermore, we verify that the SUPER scheme can be used to generate single photons. For this, we choose excitation parameters yielding maximal occupation (indicated by the red dot in Figure 3), that is, we set Δ_1_ = −4.9 meV, *I*_1_ = 0.5, Δ_2_ = −10.6 meV, and *I*_2_ = 0.64. We then measure the single-photon characteristics in a Hanbury Brown and Twiss (HBT) setup. The results are displayed in Figure 4b. We achieve a *g*^(2)^(0) = 0.06(1), which is a very promising result toward the goal of producing high quality single photons, considering that the experiments are performed at *T* = 8 K. Under s-shell resonant excitation, we observed a *g*^(2)^(0) = 0.016^30^ on the same quantum dot. We find that the recorded *g*^(2)^(0) under SUPER is slightly higher than under s-shell resonant excitation due to the laser scattering background, considering that the excitation power in SUPER is much higher compared to s-shell resonant excitation. We are, however, confident that scattered laser light can be suppressed better with moderate experimental effort.

It is also worthwhile to discuss SUPER in comparison to existing resonant or near-resonant excitation schemes.^7^ Among those, coherent schemes include Rabi rotations,^2,3,31,32^ chirped excitations exploiting the adiabatic rapid passage effect,^5,6,33−37^ and dichromatic excitation.^8,9,38^ Preparation of the exciton state can also be achieved by TPE to the biexciton state followed by a timed stimulation of the biexciton-to-exciton transition.^39−41^ Although all of these schemes have their own advantages and disadvantages, the superiority of SUPER is that it circumvents the need for polarization filtering and is quite flexible regarding the chosen detuning values. While polarization filtering is also uncalled for in the dichromatic scheme, a clear advantage of SUPER is that both pulses are red-detuned and therefore no higher-lying states of the quantum dot will be directly addressed.

Another group of state-preparation schemes are phonon-assisted processes,^19,27,42−46^ which require an additional particle, the phonon, to function. Hence, those schemes are incoherent, which might be disadvantageous when preparing superposition states. In addition, the laser pulses in phonon-assisted schemes are blue-detuned. Therefore, SUPER might also be a viable alternative to these schemes.

In conclusion, this work demonstrates that a red-detuned pulse pair can populate the exciton state in a quantum dot relying on the SUPER mechanism. This is astonishing given that a single pulse at these far detunings does not lead to a population inversion. In particular, the excitation below the absorption edge removes the stringent need of polarization filtering. Our simple yet elegant implementation of this new technique through amplitude shaping contributes toward an effortless method for generating high-purity single photons.

## Supporting Information Available

Experimental details; background estimation; normalization of the pulse intensities; verification of the two-pulse effect; optimizing the first pulse intensity; lifetime and intrinsic line width; radiative efficiency (PDF)
