# Supplementary material for: SUPER Scheme in Action: Experimental Demonstration of Red-Detuned Excitation of a Quantum Emitter
Source: Nano Lett. 2022 Jul 6;22(16):6567–72. doi: 10.1021/acs.nanolett.2c01783 (PMC9413213; doi:10.1021/acs.nanolett.2c01783)
Supplement: Supplementary file 1 — nl2c01783_si_001.pdf [file nl2c01783_si_001.pdf]

Supporting Information for SUPER Scheme in Action: Experimental Demonstration of Red-detuned Excitation of a Quantum Emitter

Yusuf Karli ,[*,†,‖] Florian Kappe,[†,‖] Vikas Remesh,[†] Thomas K. Bracht,[‡] Julian Münzberg,[†] Saimon Covre da Silva,[¶] Tim Seidelmann,[§] Vollrath Martin Axt,[§] Armando Rastelli,[¶] Doris E. Reiter,[‡,⊥] and Gregor Weihs[†]

†*Institut für Experimentalphysik, Universität Innsbruck, Innsbruck, 6020, Austria*

‡*Institut für Festkörpertheorie, Universität Münster, Münster, 48149, Germany*

¶*Institute of Semiconductor and Solid State Physics, Johannes Kepler University Linz, Linz, 4040, Austria*

§*Theoretische Physik III, Universität Bayreuth, Bayreuth, 95440, Germany*

‖*These two authors contributed equally*

⊥*Current affiliation: Condensed Matter Theory, Department of Physics, TU Dortmund, Dortmund, 44221, Germany*

Experimental details

The home-built $4f$ pulse shaper constructed in folded (reflection) geometry includes a programmable, liquid crystal on silicon spatial light modulator (SLM-128-A-VN, CRi Inc., 128 pixels, single mask). Here, the incoming laser beam first hits a grating and disperses,

before being focused onto the SLM that is kept at the Fourier plane of a curved mirror. Effectively, each pixel of the SLM holds a narrow spectral region of the dispersed laser beam. A flat mirror kept behind the SLM that is tilted slightly on the vertical axis, provides vertical displacement to pick up the reflected beam above the incoming path.

Precise amplitude shaping in the $4f$ shaper relies on the unique frequency to spatial conversion at the Fourier plane of the concave lens. Therefore, an *a priori* knowledge on the resolution of the $4f$ shaper is very important.

A beam with a finite width of W on the grating plane will have an angular spread of $\frac{\lambda_0}{W}$, where λ_0 is the central wavelength. This wavelength-dependent spread determines the resolution of the $4f$ shaper, and for a focal length f of the curved mirror, this corresponds to $\delta x = \frac{f\lambda_0}{W}$. In our setup, W (measured at $\frac{1}{e^2}$ at the grating plane) is $7.7\,\mathrm{mm}$ and $f = 500\,\mathrm{mm}$ and $\lambda_0 = 802\,\mathrm{nm}$. For the incidence angle of $54.5\,^\circ$ and a grating period d of $\frac{1}{1800}$ l/mm, the angular dispersion coefficient of the grating is given by $\alpha = \frac{\lambda_0^2}{cd\cos(\theta_d)}$ where θ_d is the first order diffraction angle for λ_0. This leads to $\delta\lambda = 0.045\,\mathrm{nm}$ and $\delta x = 51.7\,\mu\mathrm{m}$. The pixel width of our SLM is $\approx 100\,\mu\mathrm{m}$, implying that every pixel in the $4f$ shaper holds $\approx 0.09\,\mathrm{nm}$ (or $\approx 0.17\,\mathrm{meV}$), which is the resolution of the $4f$ shaper.

The distance traveled by the laser beam within the shaper amounts strictly to $4f$, to ensure that the laser pulses leave the pulse shaper free of dispersion[1]. We verify this condition through a nonlinear measurement of the pulse duration by an autocorrelator (PulseCheck, APE GmBH). Next, we calibrate the relationship between the applied voltage drive level and the resultant phase retardance of the liquid crystals based on the standard procedure[2]. The achievable amplitude modulation is proportional to the cosine of the retardance of the liquid crystal cells. This enables the generation of a phase-locked pair of pump pulses with arbitrary amplitudes and detunings Δ_1 and Δ_2 as described in the main text.

The quantum dots chosen for this experiment are grown by the droplet epitaxy technique[3,4]. This process results in dots grown in random locations on the sample. To locate

a bright quantum dot, we illuminate the sample with an above band laser beam ($532\,\mathrm{nm}$, Thorlabs) and optimize the position with the three-axis piezo stack. The focal spot size and the density of the dots in the sample ensure that no two dots are excited in a single excitation spot.

Background estimation

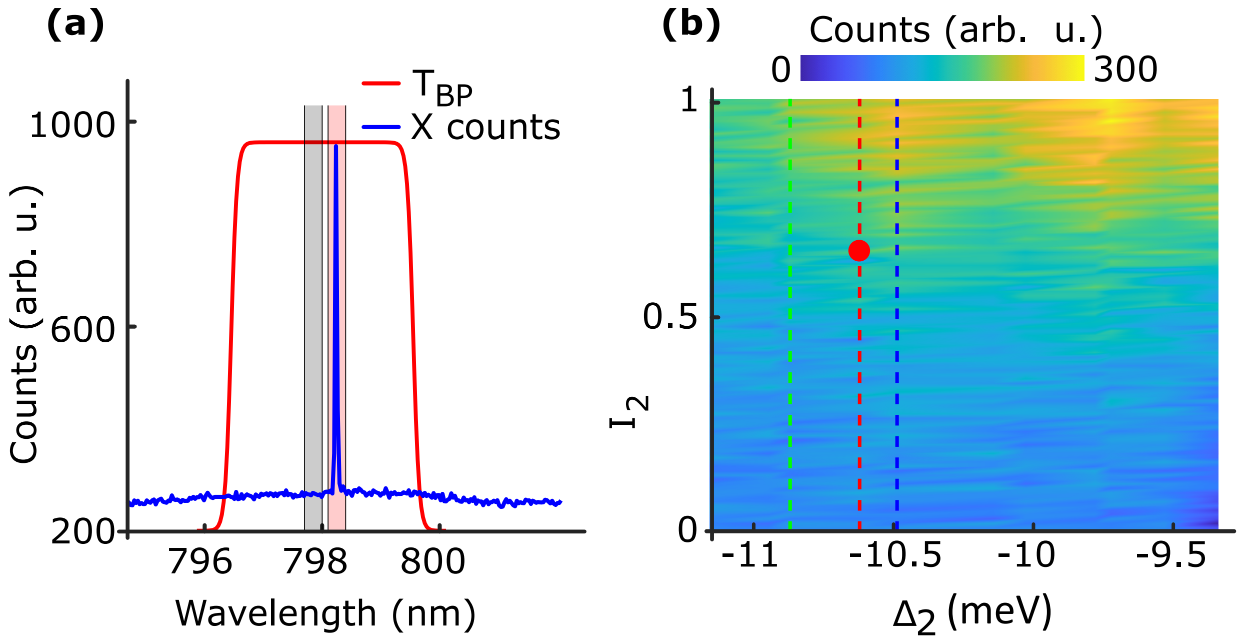

Figure S1: **Data processing and background estimation:** (a) A representative exciton emission spectrum (blue solid line) measured in the experiment; transmission spectrum of the bandpass filter used (solid red line) . The shaded red (grey) area is used for extracting the exciton (background) photon counts. (b) Background contribution in the $I_2 - \Delta_2$ scan, cf. Figure 3. Red dot denotes the parameters for photon quality measurements, cf. Figure 4 in discussion section , yielding a $9(1)\,\%$ background contribution.

In the experiments, the emitted light from the quantum dot is sent to the spectrometer, after passing through a bandpass filter with FWHM of $3\,\mathrm{nm}$ at a central wavelength of $798\,\mathrm{nm}$ (red solid curve in Figure S1 (a)). An exemplary emission spectrum shown as blue solid curve in Figure S1 (a) suggests a wavelength-independent background around the exciton emission line, which we attribute to scattered laser background under high

power illumination. For the results displayed in Figure 3, the background contribution is estimated from the integrated photon counts in the grey-shaded window and is then subtracted from the integrated photon counts in the red-shaded window (Figure S1 (a)). The shaded windows correspond to $0.3\,\mathrm{nm}$ spectral width, determined by the FWHM of the notch filter through which the exciton photons are sent to the SNSPD. In Figure S1 (b) we show the variation in the background corresponding to the results displayed in Figure 3. On comparison, we estimate a $9(1)\,\%$ contribution of background noise in the photon quality measurements (red dot in Figure S1).

Normalization of the pulse intensities

For the experiment, the pulse pairs (with various Δ_2 and I_2) are amplitude-shaped from the broadband laser spectrum as explained in the main text. This means that the individual intensities of the detuned pair depend on their spectral locations in the unshaped Gaussian intensity spectrum. In other words, the maximum of I_2 will not be the same for different Δ_2. We therefore apply a correction procedure to normalize I_2. We apply Gaussian fits to the measured spectra for various detunings Δ_2, compute the integrated spectral intensities, and obtain $I_{2,corr}$ with respect to a maximum transmission of $I_2 = 1$ for $\Delta_2 = -9.4\,\mathrm{meV}$ (see Figure S2, inset).

We then normalize I_2 with relative pulse intensities based on a fifth order polynomial fit, as shown in Figure S2. Based on this fit function, we calibrate transmissivities $I_1(I_2)$ with respect to the measured power values $P_1^{\mathrm{avg}}(P_2^{\mathrm{avg}})$, as summarized in the table 1. The results presented in Figure 3(a) and 3(c) will, as a result, get modified to S2(c) and (d), where the I_2 axis is replaced by P_2^{avg} values.

Table 1: Average power of the pulses $\Delta_1 = -4.9\,\mathrm{meV}$ and $\Delta_2 = -10.6\,\mathrm{meV}$, measured at the cryostat entrance window.

$\boldsymbol{I_1}$	$\boldsymbol{P_1^{\mathrm{avg}}}$ (μW)	$\boldsymbol{I_2}$	$\boldsymbol{P_2^{\mathrm{avg}}}$ (μW)
0	0.4	0	0.7
0.5	15.5	0.65	10.9
1	34.2	1	18.6

Table 2 shows the polynomial fit coefficients.

Table 2: Polynomial coefficients extracted from data presented in figure S2.

p_5	p_4	p_3	p_2	p_1	p_0
-1.83	3.39	-2.36	1.57	0.19	0.04

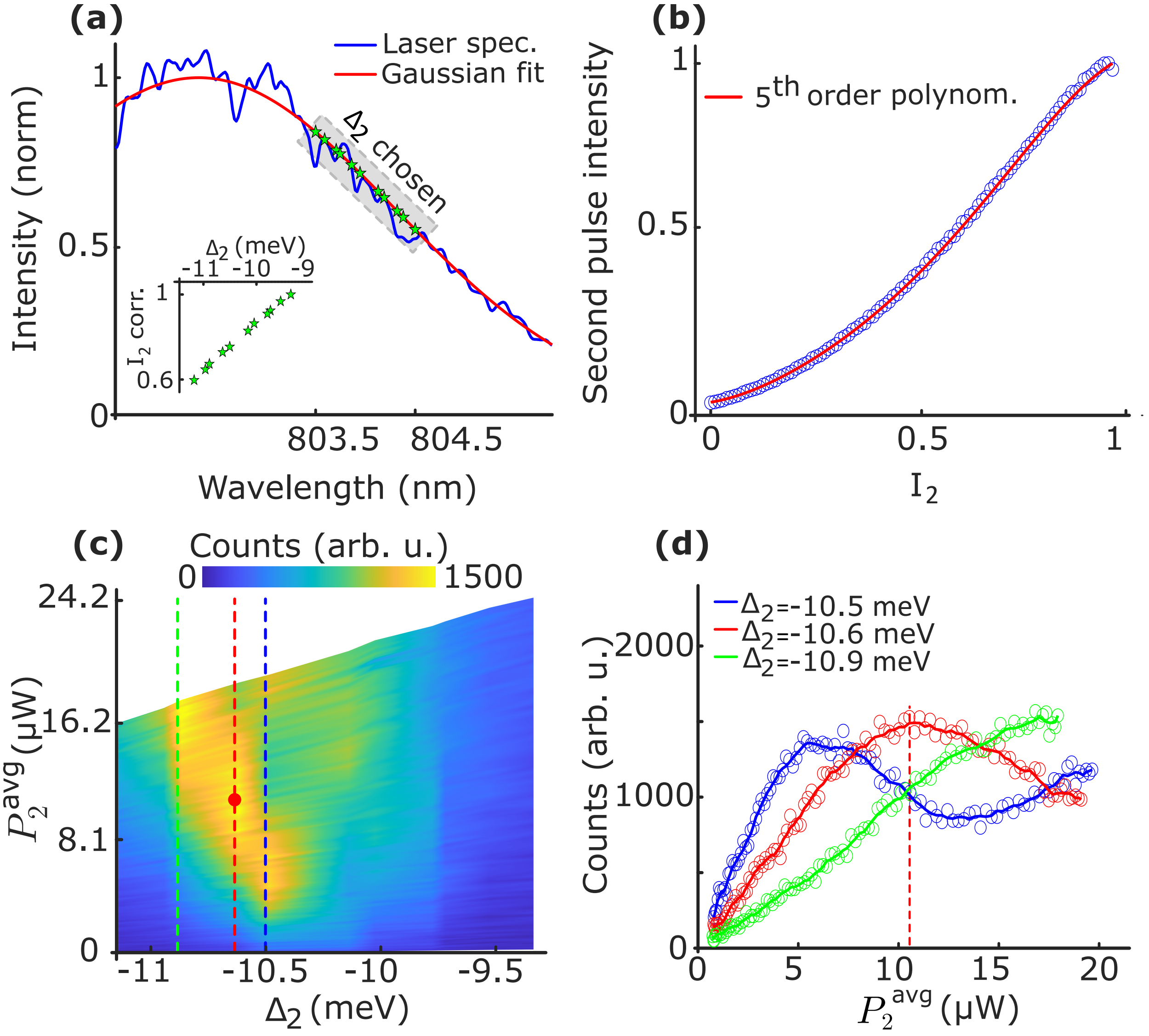

Figure S2: **Corrected transmissivities of individual pulses:** (a) Measured excitation laser spectrum (blue solid line), fit with a Gaussian function (red solid line) where the eleven chosen Δ_2 values for the experiments are indicated (green stars, grey shaded and inset). (b) Transfer function to convert I_2 to the normalized intensity of the second pulse (blue circles). Red solid line is a fifth order polynomial fit. (c) and (d) Corrected Figure 3(a) and 3(c) for I_2 values calibrated for P_2^{avg} of the second pulse. The first pulse intensity ($I_1 = 0.5$) is $15.5\,\mu$W.

Verification of the two-pulse effect

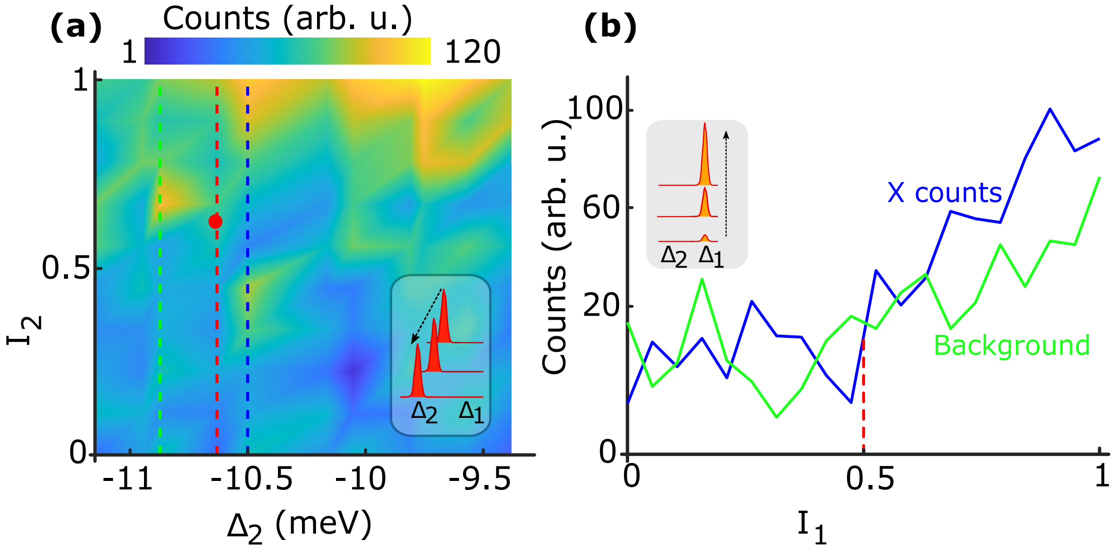

Figure S3: **Single pulse excitation:** (a) Measured photon counts at exciton-emission energy with ($I_1 = 0$) for a $I_2 - \Delta_2$ scan as in Figure 3. Note the change in colour scale showing that only negligible photon counts are recorded. (b) Measured photon counts as in (a) without the second pulse ($I_2 = 0$) at $\Delta_1 = -4.9\,\text{meV}$ and $I_1 = 0 \ldots 1$. Red dashed line shows the experimental conditions $I_1 = 0.5$ for all the results displayed in the main text.

To verify that the measured exciton population is the effect of the two-pulse excitation, we perform control experiments. For this, we first set $I_1 = 0$, and perform the $I_2 - \Delta_2$ scan, as described in the main text. We observe that, as shown in Figure S3(a), the measured exciton counts are insignificant. Next, we set $I_2 = 0$ (which implies there is no Δ_2 scan), and simply perform a I_1 scan, recording the exciton counts. The results are displayed in Figure S3(b). The integrated exciton counts vary in accordance with the rise in the background, indicating yet again the absence of the SUPER effect. The red dashed line in Figure S3(b) denotes the experimental conditions for the results displayed in Figure 3, asserting that the measured exciton counts are only due to the two-pulse excitation. Nonetheless, for $I_1 > 0.8$, we observe a minor increase in the exciton counts, which we attribute to the substrate luminescence. Thus, for the below absorption edge, SUPER excitation of the quantum dot, we strictly require two detuned pulses.

Optimizing the first pulse intensity

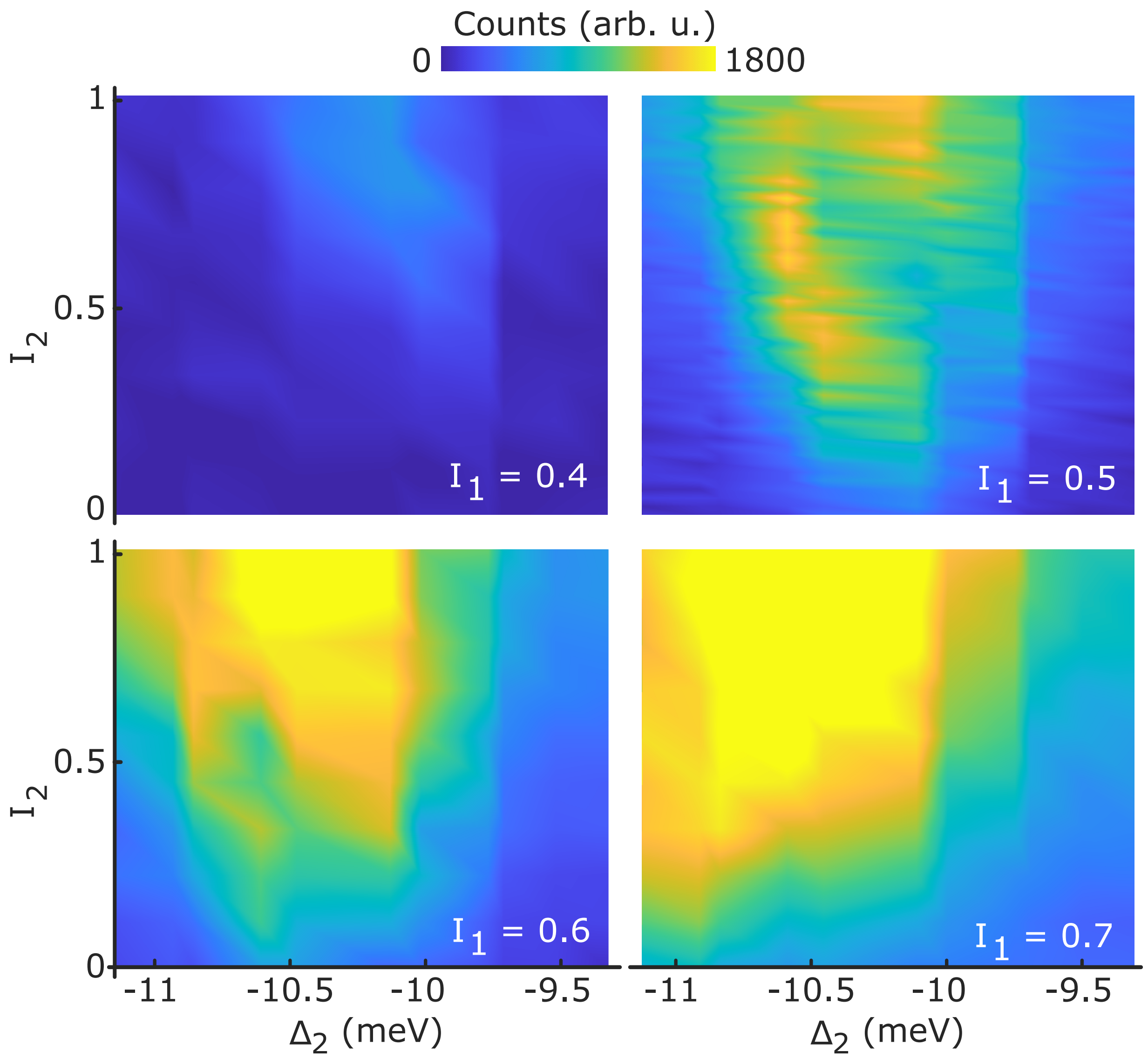

Figure S4: **Variation of exciton counts with I_1:** Measured population of the exciton state as function of Δ_2 and I_2 for fixed intensities of the first pulse $I_1 = 0.4, 0.5, 0.6, 0.7$ (cf. Figure 3(a)).

Here, we investigate the influence of the first pulse intensity I_1 in the SUPER scheme. At first, we set $I_1 = 0.4 \ldots 0.7$, keeping $\Delta_1 = -4.9\,\mathrm{meV}$ fixed. We then perform the $I_2 - \Delta_2$ scan as in Figure 3(a). Note that for the data displayed in Figure 3(a) we chose $I_1 = 0.5$. For $I_1 < 0.4$, we measure only a modest exciton photon count, indicating that the exciton

state is not efficiently populated. At higher I_1 values, we do not observe the oscillatory trend as in Figure 3(c), but the increase in photon counts with I_2, indicates that the SUPER excitation takes place here too.

Lifetime and intrinsic linewidth

We validate the nature of the emission by measuring the decay dynamics. To this end, we send the emitted photons to the SNSPD for the time-correlated single-photon counting measurement with $20\,\mathrm{ps}$ temporal resolution. An internal photodiode within the excitation laser serves as the start signal, while single photon clicks act as stop signals. The resulting time-correlated histogram is fit with an exponential function, which leads to a computed lifetime of $165.1(6)\,\mathrm{ps}$. This is in excellent agreement with our results under resonant excitation of the same quantum dot[5] at 8K. From the computed lifetime value we obtain an intrinsic linewidth ($1/T_1$) of 0.965 GHz or 3.95 μeV. Under TPE, we have also observed similar lifetime values. Additionally, from preliminary measurements of coherence length/coherence time in a Michelson interferometer[6–10] at 8K under TPE, we have measured a coherence time (T_2) of 173 ps, which leads to a linewidth of 1.84 GHz or 7.6 μeV. This yields a pure dephasing time T_2* of 363.63 ps, however, because such linewidth measurements on quantum dots make sense only at very low temperatures (to limit pure dephasing, which is relevant in the large dots used here), which we leave for future work.

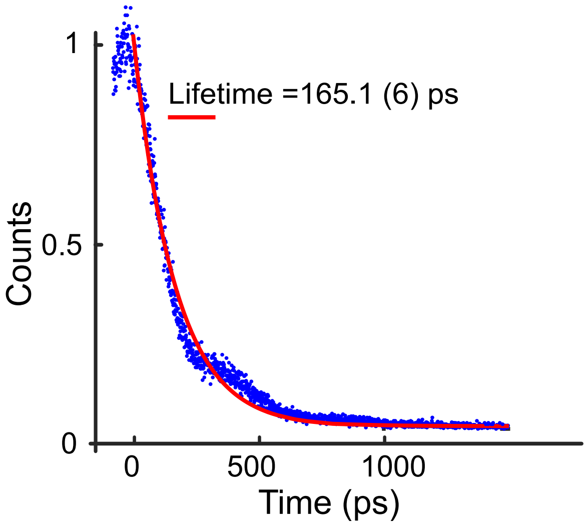

Figure S5: Radiative lifetime of the exciton: Measured decay dynamics of the exciton emission under SUPER, leading to a computed lifetime of $165.1(6)\,\mathrm{ps}$.

Radiative efficiency

In semiconductor quantum dots, the diffusion of charge carriers from its vicinity results in an on-off modulation in the emitted light, usually called blinking. As a result, the long-term g(2) measurement shows an exponential drop in the recorded photon counts[11], effectively limiting the radiative efficiency of the quantum dot. Here we show the results of HBT measurement on a long timescale, under SUPER. The on-time fraction of the quantum dot is ≈ 0.33 under SUPER and 0.30 under TPE[12] and 0.36 under resonant excitation[5]. Notably, for resonant excitation and TPE, this factor also depends on a supportive above-band or white-light illumination[13].

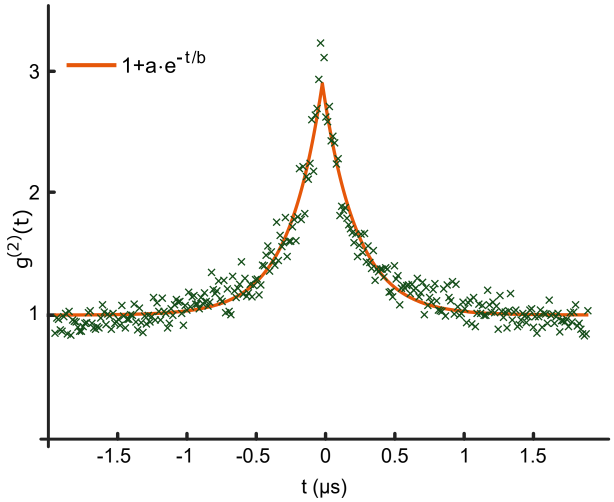

Figure S6: Radiative efficiency: Long-timescale $g^{(2)}(0)$ measured on the quantum dot under SUPER. The inverse of the $g^{(2)}(0)$ denotes the on-time fraction of the quantum dot, which we observe as 0.33.

References

(1) Weiner, A. M. Femtosecond pulse shaping using spatial light modulators. *Rev. Sci. Instrum.* **2000**, *71*, 1929–1960.

(2) Monmayrant, A.; Weber, S.; Chatel, B. A newcomer's guide to ultrashort pulse shaping and characterization. *J. Phys. B At. Mol. Opt. Phys.* **2010**, *43*, 103001.

(3) Huber, D.; Reindl, M.; Huo, Y.; Huang, H.; Wildmann, J. S.; Schmidt, O. G.; Rastelli, A.; Trotta, R. Highly indistinguishable and strongly entangled photons from symmetric GaAs quantum dots. *Nat. Commun.* **2017**, *8*, 15506.

(4) da Silva, S. F. C.; Undeutsch, G.; Lehner, B.; Manna, S.; Krieger, T. M.; Reindl, M.; Schimpf, C.; Trotta, R.; Rastelli, A. GaAs quantum dots grown by droplet etching epitaxy as quantum light sources. *Appl. Phys. Lett.* **2021**, *119*, 120502.

(5) Münzberg, J.; Draxl, F.; da Silva, S. F. C.; Karli, Y.; Manna, S.; Rastelli, A.; Weihs, G.; Keil, R. Fast and efficient demultiplexing of single photons from a quantum dot with resonantly enhanced electro-optic modulators. **2022**, 2203.08682, arXiv(Quantum Physics), `http://arxiv.org/abs/2203.08682`, (accessed March 16, 2022).

(6) Reimer, M. E.; Bulgarini, G.; Fognini, A.; Heeres, R. W.; Witek, B. J.; Versteegh, M. A.; Rubino, A.; Braun, T.; Kamp, M.; Höfling, S., et al. Overcoming power broadening of the quantum dot emission in a pure wurtzite nanowire. *Phys. Rev. B* **2016**, *93*, 195316.

(7) Jayakumar, H.; Predojević, A.; Kauten, T.; Huber, T.; Solomon, G. S.; Weihs, G. Time-bin entangled photons from a quantum dot. *Nat. Commun.* **2014**, *5*, 4251.

(8) Gold, P.; Thoma, A.; Maier, S.; Reitzenstein, S.; Schneider, C.; Höfling, S.; Kamp, M. Two-photon interference from remote quantum dots with inhomogeneously broadened linewidths. *Phys. Rev. B* **2014**, *89*, 035313.

(9) Zwiller, V.; Aichele, T.; Benson, O. Single-photon Fourier spectroscopy of excitons and biexcitons in single quantum dots. *Phys. Rev. B* **2004**, *69*, 165307.

(10) Reindl, M.; Jöns, K. D.; Huber, D.; Schimpf, C.; Huo, Y.; Zwiller, V.; Rastelli, A.; Trotta, R. Phonon-assisted two-photon interference from remote quantum emitters. *Nano Lett.* **2017**, *17*, 4090–4095.

(11) Davanço, M.; Hellberg, C. S.; Ates, S.; Badolato, A.; Srinivasan, K. Multiple time scale blinking in InAs quantum dot single-photon sources. *Phys. Rev. B* **2014**, *89*, 161303.

(12) Schimpf, C.; Manna, S.; Da Silva, S. F. C.; Aigner, M.; Rastelli, A. Entanglement-based quantum key distribution with a blinking-free quantum dot operated at a temperature up to 20 K. *Adv. photonics* **2021**, *3*, 065001.

(13) Schimpf, C.; Reindl, M.; Klenovský, P.; Fromherz, T.; Silva, S. F. C. D.; Hofer, J.; Schneider, C.; Höfling, S.; Trotta, R.; Rastelli, A. Resolving the temporal evolution of line broadening in single quantum emitters. *Opt. Express* **2019**, *27*, 35290–35307.
